# Dr. Tebault’s Paper

**Published:** 1899-05

**Authors:** 


Dr. Tebault's Paper.—We call attention to the paper by Dr. Tebault, Surgeon General United Confederate Veterans Association, in the department of "Abstracts and Selections" in this issue. Any statement emanating from so distinguished and capable an observer is entitled to much consideration; he is a practitioner of a great many years,—I will not say *how* many; and was a surgeon in the Confederate army. His experience with smallpox in those trying times resulted in the expedient of inoculating the exposed persons with a modified lymph from the vesicles, as stated, and the results were most satisfactory. At that time it was impossible to get reliable vaccine matter; in fact, to get any kind of vaccine matter; and it was the custom to take the "scab" from the arm of a person who had been vaccinated and use it upon others. In this way doubtless many persons were inoculated with disease, and it was quite common to see terrible results follow such practice. Under those circumstances Dr. Tebault's plan was most commendable, and far preferable to the one of using humanized virus which had been so extensively "humanized." But now, at least in this country, in these "piping times of peace," when a glycerized lymph, in sterilized and sealed glass tubes, propagated and collected under the most scrupulous antiseptic precautions, can be had in all cities, it is no longer necessary to resort to any expedient, or to substitute inoculation for vaccination, unless under exceptional circumstances; for instance, in cases where, after repeated trials, the vaccine has failed to "take" (and we have all seen such cases), and the "doctor" has sometimes told the party that it was "a sign that he couldn't have smallpox." In those cases the use of Dr. Tebault's plan is a valuable resource and may be adopted with benefit. Again, in sections more or less remote, on the occurrence of a case of smallpox, and it is necessary to quickly protect those who have been exposed or will be exposed; where fresh and reliable vaccine cannot be had, or had without delay, physicians will do well to remember that the lymph from a vesicle, rubbed up with fresh cow's-milk, will make an excellent protective. We commend Dr. Tebault's article to the attention of our readers as being especially timely these days of widespread prevalence of smallpox, in rural districts especially.

